# Dipeptidyl Peptidase (DPP)-IV Inhibitors with Antioxidant Potential Isolated from Natural Sources: A Novel Approach for the Management of Diabetes

**DOI:** 10.3390/ph14060586

**Published:** 2021-06-18

**Authors:** Anand-Krishna Singh, Dhananjay Yadav, Neha Sharma, Jun-O Jin

**Affiliations:** 1 Shri Vaishnav Institute of Science, Shri Vaishnav Vidyapeeth Vishwavidyalaya, Indore, Madhya Pradesh 453555, India; 2 Department of Medical Biotechnology, Yeungnam University, Gyeongsan 38541, Korea; dhanyadav16481@gmail.com; 3 School of Life Sciences, Devi Ahilya University, Indore, Madhya Pradesh 452001, India; nehasharma.908@gmail.com; 4 Research Institute of Cell Culture, Yeungnam University, Gyeongsan 38541, Korea

**Keywords:** dipeptidyl peptidase-IV, glucagon-like peptide-1, hyperglycemia, incretin, antioxidant

## Abstract

Type 2 diabetes mellitus (T2DM) is characterized by hyperglycemia that is predominantly caused by insulin resistance or impaired insulin secretion, along with disturbances in carbohydrate, fat and protein metabolism. Various therapeutic approaches have been used to treat diabetes, including improvement of insulin sensitivity, inhibition of gluconeogenesis, and decreasing glucose absorption from the intestines. Recently, a novel approach has emerged using dipeptidyl peptidase-IV (DPP-IV) inhibitors as a possible agent for the treatment of T2DM without producing any side effects, such as hypoglycemia and exhaustion of pancreatic β-cells. DPP-IV inhibitors improve hyperglycemic conditions by stabilizing the postprandial level of gut hormones such as glucagon-like peptide-1, and glucose-dependent insulinotropic polypeptides, which function as incretins to help upregulate insulin secretion and β-cell mass. In this review, we summarized DPP-IV inhibitors and their mechanism of inhibition, activities of those isolated from various natural sources, and their capacity to overcome oxidative stress in disease conditions.

## 1. Introduction

Diabetes mellitus, commonly identified as diabetes, is a cluster of metabolic disorders that are distinguished by high blood glucose levels over a prolonged period of time and associated with inadequate production of insulin by the pancreatic β-cells and insulin resistance. Insulin aids in the uptake of glucose into the cells of the body, and is therefore required for the utilization of glucose from digested foods. Diabetes is characterized by chronic hyperglycemia with impaired carbohydrate, fat, or protein metabolism due to impaired insulin secretion, insulin resistance, or both. The effects of diabetes include long-term injury, dysfunction, and failure of various organs. Diabetes monitoring has changed substantially during the past 70 years, and therapeutic options today are relatively more effective and less costly than those of the past. The current pharmacological drugs used to treat diabetes have resulted in drastic reductions in morbidity and mortality. There are now more than 11 distinct categories of medications available for the treatment of hyperglycemia in patients with diabetes. These medicinal drugs have been developed over the past 90 years [1].

Currently treatments of type 2 diabetes mellitus (T2DM) include oral hypoglycemic agents, and injectable agents. Pharmacologic treatments for T2DM allow better control of glycemic conditions and hypertension, and reduce blood lipid concentrations [2,3]. New treatment approaches for diabetic complications would improve if the molecular mechanisms of diabetes pathology and related β-cell pathway genetic defects could be understood more clearly [4]. The occurrence of T2DM in different regions of the world shows a vast pattern in urban and rural areas. As per the World Health Organization (WHO) and the 9th edition of the International Diabetes Federation (IDF), diabetes is the world’s seventh leading cause of death, and every six seconds a person dies from diabetes. Roughly, more than 463 million people of the world’s population had diabetes mellitus in 2019, which is much higher than an earlier estimate of 382 million people in 2013, and the number of patients with diabetes is expected to rise to 700 million by 2045 [5,6].

**Figure 1:**
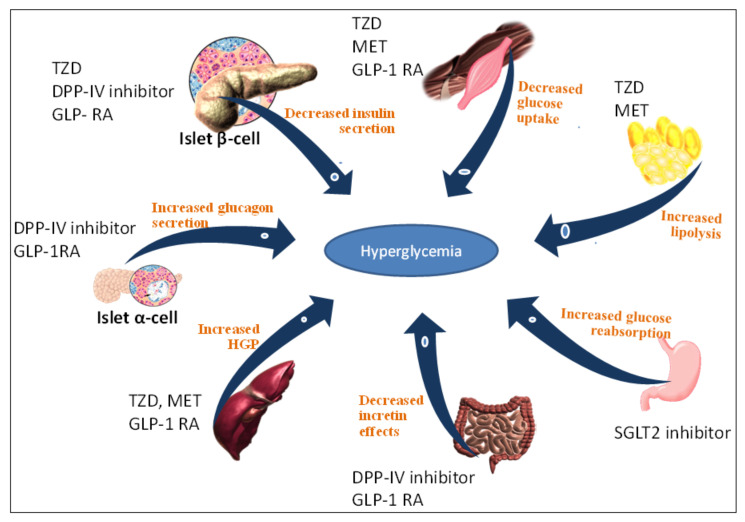
Different approaches for the treatment of hyperglycemia recommended to type 2 diabetes mellitus patients. Abbreviations: thiazolidinedione (TZD), metformin (MET), dipeptidyl peptidase-IV inhibitor (DPP-IV), glucagon-like peptide-1 receptor antagonist (GLP-I RA), sodium-glucose co-transporter-2 inhibitor (SGLT2).

Current existing therapies are insufficient due to several reasons, such as poor adherence in patients, lack of efficacy and side effects. Several medicines are currently being used in clinics such as metformin, sulfonylurea, thiazolidinedione, and sodium-glucose co-transporter-2 inhibitor, but they have side effects including insulin resistance, hypoglycemia, hypothyroidism, weight gain, abdominal pain, obesity, and atherosclerosis [7,8,9]. Figure 1 shows the different pathophysiological reasons for hyperglycemia development, such as increased hepatic glucose production, increased glucagon secretion, decreased pancreatic α-cell and β-cell insulin production, decreased muscle glucose uptake, increased glucose reabsorption, and decreased incretin effects.

The usual manifestation of untreated diabetes is weight loss, increased urination (polyuria), excessive thirst (polydipsia), increased hunger (polyphagia), and blurred vision [10]. Lack of proper treatment can lead to certain complications such as nephropathy which may cause renal failure and increases the risk of foot ulcers, retinopathy with blindness, and autonomic dysfunction, including sexual dysfunction [11]. Diabetes complications and pathophysiology are usually accompanied by immoderate production of reactive oxygen species by mitochondria, the respiratory chain, non-enzymatic glycation, and metabolic disorders. Increased reactive oxygen species (ROS) production due to the overproduction of free radicals or decreased levels of enzymes, such as superoxide dismutase (SOD), catalase, and glutathione reductase, leads to impaired antioxidant defenses antioxidant defenses that are needed to inhibit or scavenge the free radicals that are generated by metabolic disorders and oxidative stress and promote good health [12]. These molecules significantly reduce the damage caused by oxidant molecules by scavenging or neutralizing free radicals before they can attack cells. These mechanisms prevent damage to lipids, proteins, enzymes, carbohydrates, and nucleic acids. A novel approach to control diabetes is based on the use of glucagon-like peptide-1 (GLP-1), an incretin/gut hormone, which has been shown to reduce postprandial and fasting glycemia in type 2 diabetes mellitus (T2DM) [13,14]. However, GLP-1 is rapidly degraded in blood plasma by the dipeptidyl peptidase-IV enzyme (DPP-IV). Therefore, DPP-IV inhibitors might be resulting in the prolongation of the half-life of GLP-1 by inhibiting DPP-IV and the increase in GLP-1 levels. Hence, the mode of action relies on inhibiting the degradation of GLP-1 and increase in GLP-1 levels in the blood that lead to an increase in the secretion of insulin from the pancreas in the body and maintains the glucose homeostasis. In this review, we discussed the traditional medicinal plants and animal by-products that contain naturally occurring DPP-IV inhibitors and their antioxidant properties, for the treatment of T2DM.

## 2. T2DM Treatment Strategy Based on DPP-IV Serine Protease Inhibition

**Figure 2:**
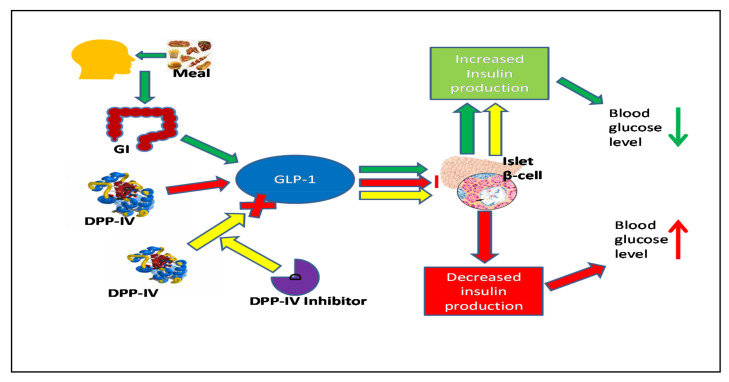
Diagrammatic representation of the mechanism of DPP-IV and potential role of protecting incretin hormone degradation by DPP-IV enzyme to maintain the homeostatic blood glucose level. Abbreviations: gastrointestinal tract (GI), dipeptidyl peptidase-IV inhibitor (DPP-IV), glucagon-like peptide-1 (GLP-1).

Dipeptidyl peptidase-IV (DPP-IV) enzyme inhibitors have provided a unique therapeutic perspective for the treatment of T2DM [15]. Furthermost, mechanisms based on gut hormones, glucagon-like peptide-1 (GLP-I), glucose-dependent insulinotropic-polypeptide (GIP), and gastric inhibitory polypeptide, have been shown to reduce postprandial and fasting glycemia in patients with T2DM and provide therapeutic potential for T2DM. The role of these hormones has significant importance in β-cell survival, and in increasing β-cell mass and insulin production. They also regulate glucose homeostasis by enhancing insulin levels in the blood [16,17,18]. However, GLP-1 and GIP are constantly broken down in blood plasma by DPP-IV, leading to their inactivation and reduction in their half-life from five minutes to two minutes. Hence, they no longer promote insulin secretion from β-cells. Therefore, DPP-IV enzyme inhibitors play a significant role in improving the half-life of incretin hormones, i.e., by prolonging their blood circulation via DPP-IV inhibition. Consequently, preventing the cleavage of gut incretins GLP-1 and GIP has drawn attention as a beneficial management strategy for T2DM. Figure 2 briefly represents the mechanism of DPP-IV inhibitors. After consuming meals, GLP-1 incretin hormone is released in the gastrointestinal (GI) tract and cleaved by the DPP-IV enzyme. However, if a DPP-IV inhibitor is present in the blood, it protects GLP-1 cleavage by inhibiting DPP-IV enzyme and thereby regulates blood glucose levels by promoting insulin secretion from the pancreas. Certain FDA-approved drugs such as vildagliptin and sitagliptin are competitive, orally active, and fully reversible DPP-IV inhibitors. DPP-IV inhibitors are also the first incretin-based class of antidiabetic drugs to gain regulatory consent for the treatment of T2DM [19,20,21].

Medicines based on incretin-based mechanisms have been proposed as treatments for T2DM and include DPP-IV inhibitors and GLP-1 antagonists. DPP-IV inhibition has a unique mechanism that is associated with a lack of hypoglycemia, weight loss, and minimal side effects. These characteristics may facilitate better therapeutic treatment and help to achieve our aim. Table 1 summarizes synthetic DPP-IV inhibitors that have been approved by the FDA.

## 3. DPP-IV Structure and Background

DPP-IV is classified as a serine protease with a serine, histidine, and aspartic acid catalytic triad of amino acids. The DPP-IV enzyme (DPP-IV, EC 3.4. 14. 5; CD26) was discovered by Hopsu-Havu and Glenner [22,23,24]. Collagen has a Gly-Pro amino acid sequence, and DPP-IV (also known as CD26) is incapable of cleaving Pro–Pro or Pro–Hyp bonds; thus, cleavage frequently follows the Gly–Pro sequence in collagens. Notably, the physiological functions of DPP-IV have remained unclear for many years [25,26]. Exo-peptidases that cleave N-terminal or C-terminal amino acid residues from peptides and proteins are known as amino- and carboxy-peptidases respectively [27].
Amino Acid (X) 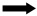 Proline (P) 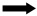 Polypeptide Chain 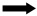 X- P + Polypeptide Chain

(N-terminus) (Penultimate amino acid)       DPP-IV (Dipeptide)

DPP-IV has the potential to cleave peptide bonds to form a penultimate amino acid proline and release proline-containing dipeptides from the N-terminus of the polypeptide chain [28,29]. It contains a small number of enzymes that are able to simply absorb proline-rich proteins. DPP-IV is generally expressed by cells in the intestinal brush border, and aids in the complete breakdown of proline-containing dietary proteins, such as casein and gluten. Some proteins that are particularly allergenic and opposed to hydrolysis by the other proteolytic enzymes have shown a variety of unfavorable food reactions and may cause enteropathic manifestations, such as celiac disease [28,30]. The soluble DPP-IV/sCD26 increases the expression of inducible nitric oxide synthase (iNOS), the production of proinflammatory cytokines in LPS treated macrophages, and is also reported to stimulate ROS production and activate the receptor for advanced glycation end product gene expression [31,32]. Additionally, sCD26 has the potential to enhance an innate immune response. THP-1 cells and monocytes were stimulated with a combination of sCD26 and LPS, which increased the expression of c-Fos, NF-kB p65, NF-kB p50, and CUX1 [33].

## 4. Distribution and Expression of the DPP-IV Enzyme & Molecular Mechanisms of the GLP-1 Receptor on the Pancreatic Endocrine System

Biological responses that inactivate bioactive peptides have been postulated for all membrane-bound proline or alanine-specific exo-peptidases. In contrast to the X–Pro amino-peptidase and Pro–X carboxy-peptidase, which showed constrained distributions, most vertebrate tissues contain the DPP-IV enzyme, although its activity diverges broadly in different tissues. DPP-IV also closely regulates blood hormones, since it is present on endothelial cells of blood vessels and circulates as a soluble enzyme in blood plasma. DPP-IV is also expressed on activated T-helper lymphocytes and subsets of macrophages. In the endocrine system, these enzymes are fully expressed in the capillary epithelia, but not frequently in parenchyma cells, except for in follicular epithelial cells of the thyroid gland or lacteal cells. DPP-IV is also expressed in specialized fibroblasts, for example in the skin and kidney. A previous study reported that this peptidase is often present at sites of physiological barriers (e.g., the blood–brain barrier) [24,34]. The GLP-1 receptor (GLP-IR) is a 456 amino acid protein with a hepta-helical G-protein coupled receptor structure, and is extensively expressed in the kidneys, lungs, heart, pancreatic islet β-cells, central nervous system, and peripheral nervous system [35,36]. Additionally, GLP-1 replenishes insulin storage by stimulating proinsulin gene expression. GLP-1R regulation is dependent on glucagon secretion, in a glucose-dependent manner; which means it reduces the risk of hyperglycemia or hypoglycemia. GLP-IR signaling activates human pancreatic and rodent exocrine cells and initiates a program towards a more endocrine-like phenotype, in relationship with increased expression of PDX-1, GLUT-2 and glucokinase genes. Some reports have shown that GLP-R agonists improve β-cell proliferation and number in islet cell lines, and counter the glucose-dependent mechanism of GLP-1 on insulin release [37,38].

## 5. Importance of Antioxidants in Overcoming Oxidative Stress in Disease and Ageing

An organism is continuously exposed to significant oxidative stress as a result of an imbalance between the antioxidative protection system and strong oxidizing substances, including free radicals. These free radicals are defined as any atoms or molecules that contain unpaired electrons in the form of ROS and reactive nitrogen species (RNS). Free radicals, mostly ROS, are produced in every compartment of cell during normal metabolism and are part of the cellular physiology system biosynthesis, cell defense, and intracellular and intercellular signaling [10]. An imbalance between antioxidants and ROS results in the formation of free radicals that lead to cellular damage. There is sufficient support in favor of the role of free radicals in various diseases, such as cardiovascular disease, diabetes, cancer, autoimmune disorders, neurodegenerative diseases, and aging [39,40,41,42,43]. Antioxidants act as catalysts in the body to scavenge free radicals and suppress the damage caused by ROS and RNS. ROS includes a combination of the superoxide anion (O_2_^−^), hydroxyl (OH^−^), hydroperoxyl (OOH^−^), peroxyl (ROO·), and alkoxyl (RO·) radicals, non-free radicals include hydrogen peroxide (H_2_O_2_), hypochlorous acid (HOCl), and RNS mainly include nitric oxide (NO·), peroxynitrite (ONOO·) nitrogen dioxide (NO_2_), ozone (O_3_), and singlet oxygen (O_2_) [44]. ROS and RNS play important roles in normal physiological processes including cellular life cycle/death, protection from pathogens, and various cellular signaling pathways. Furthermore, oxidative stress also contributes to the accumulation or enhancement of damage to macromolecules and cell organelles, including mitochondria and the Golgi body [45,46,47]. The imbalance of ROS/RNS and antioxidants leads to oxidative stress in the body. Oxidative stress also generates free radicals and damages cells and tissues.

Antioxidants are mainly of two types: enzymatic and non-enzymatic. The enzymatic nature of antioxidants is basically endogenously included superoxide dismutase, catalase, glutathione peroxidase, and glutathione reductase. Non-enzymatic antioxidants include alkaloids, phenols, ascorbic acid, flavonoids, tocopherols, carotenoids, and steroids. Enzymatic antioxidants are produced by the human body or non-enzymatically obtained from natural sources [48,49].

Antioxidants that are used to treat various diseases have a broad range of natural and synthetic origins. Natural antioxidants are present in plants that are used as food, and there are also a few chemical-based antioxidant compounds, such as butylated hydroxyanisole, butylated hydroxytoluene, and tertiary butylhydroquinone, which are used as food supplements. However, these synthetic molecules have shown toxic effects, such as kidney and liver damage, and other side effects. Flavonoids and other phenolic compounds of plant origin have been reported as free radical scavengers [50,51,52]. Spices and plants have been widely used in foods since ancient times for good odor, flavor, color, and preservative. Nowadays, it is clear that they contain antioxidants and delay the oxidation of lipids in foodstuff [53]. Meanwhile, most of these antioxidants have been used as natural defenses against diseases and infections. Currently, most of the antioxidant activities of these compounds have been explored.

The investigation of plant-derived antioxidants has received much attention and affords the identification of the compounds that have the potential to scavenge free radicals generated by diseases or oxidative stress. The most important classes of phytochemicals to pharmacologists, active flavonoids, alkaloids, and phenolic groups, have been extensively studied as treatments for various health conditions, and the properties of interest include anti-cancer, anti-inflammatory, analgesic, anti-microbial, ulcerogenic, anti-convulsant, anti-hyperlipidemic, tyrosinase inhibitor, and anti-Parkinson activities. Ample data correlate these diseases with oxidative stress and focus on the antioxidant activity of these and other active phytochemicals in vitro and in vivo [54,55]. Recently, polyphenolic molecules have drawn greater attention than any other class of natural compounds because of their significant biological roles such as anti-oxidant, anti-diabetic, ant-carcinogen, and antibacterial activities, which may lead to their recognition as potential nutraceuticals [56,57,58,59].

## 6. Biological Actions of Incretin Hormone and Peripheral Glucose Sensors

The substrate specificity of the DPP-IV enzyme along with its localization on the plasma membrane leads to the hypothesis that this protease enzyme should either take part in the final catabolism of proline-rich peptides or have a regulatory role in the inactivation of bioactive peptides. The potency with which the DPP-IV enzyme cleaves neuropeptides, peptide hormones, or cyto-chemokine substrates suggests, a role for DPP-IV enzyme activity in body fluids and cellular systems in vitro or in vivo [27,29,60]. GLP-I is secreted from gut L cells that mostly contain an alanine amino acid at the second position of GLP-1 (9-36) or GLP-1 (9-37) amides [36,61,62]. In addition, DPP-IV inactivates GIP peptides secreted by gut K-cells and rapidly clears them from kidney circulation [63].

The incretin/gut hormone has important roles and effects on glucose management and increasing insulin discharge via activation of peripheral sensors connected with increased glucose clearance. Intraportal GLP-1 leads to the dismissal of hepatic vagal afferents into the pancreatic vagal efferent and then, the uphill nerves communicate signals to the brain and passes them through neural relays to the pancreases [64,65]. Similarly, the chorisondamine inhibitors and ganglionic blockers inhibit the stimulatory effects of the portal nerve. GLP-1 helps to release insulin in rats, and evidence suggests that GLP-IR depends on neural signals emanating from the portal exchange [66]. More recent studies have revealed that GLP-1 promotes signaling to the brain to decrease insulin-stimulated glucose uptake in muscles and increase liver glycogen storage. These signals communicate through the neural pathway. Hence, synchronized discharge of digested food and GLP-1 into the portal circulation may enhance glucose approval from the autonomous mechanism of circulating incretin [67].

Continuous administration of GLP-1 lowers blood glucose levels in both fasting and postprandial diabetic conditions via inhibition of glucagon secretion, gastric emptying, and improvement of insulin release [68,69]. The consequence of a basal level of GLP-1 for control of glycaemia, which is independent of meals, and the continuous infusion of GLP-1, from midnight to early morning, causes a drastic decrease in glucose concentration during an overnight phase in diabetic conditions [70]. The role of GLP-1 on gastric empty in the human body is effectively significant and can cause noticeable meal-related glucose digression. GLP-1 decreases insulin secretion from pancreatic β-cell which also depends upon the concentration of glucose after GLP-1 administration. Some studies have revealed that GLP-1 significantly decreases blood sugar levels in patients with T2DM [71,72,73].

## 7. Mechanism of DPP-IV Inhibitors with Antioxidant Potential

Numerous studies have revealed that DPP-IV inhibition helps increase β-cell function, physiology and mass through incretin discharge. Thus, incretin causes a continuous release of insulin after meal ingestion to lower glucose levels, which is an indication of improved β-cell function [16,26,74,75]. Recently, antioxidants have played an important role in minimizing diabetes effects by scavenging the free radicals generated by oxidative stress or through dual functions that involve targeting the causes of metabolic syndromes/diseases and minimizing free radical generation. Antioxidants protect cells from harmful oxidants (ROS and RNS) by removing the oxidants and repairing the damage that antioxidants cause in the body. DPP-IV inhibitors suppressed the toll-like receptor-4 (TLR) in mononuclear cells by modulating IL-1, IL-6, and other proinflammatory cytokines [76]. DPP-IV inhibitors also suppressed the gene expression of acyl-coenzyme A (CoA): cholesterol acyltransferase/CD36 by modulating the effect of glycation end product (AGE) and inhibiting the TLR4/IRAK-4 signaling pathway by suppressing LPS-induced IRAK-4 phosphorylation and regulating Cav-1 interaction with CD26 [77,78].

**Figure 3:**
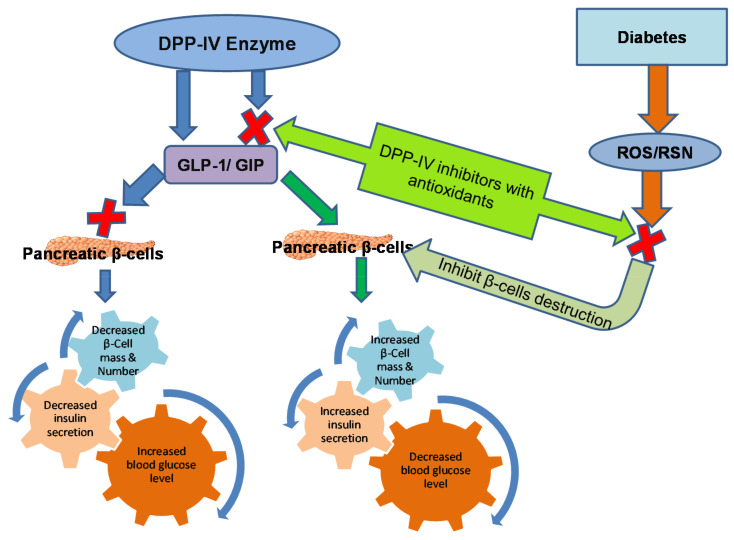
Role of DPP-IV inhibitors on GLP-1 dependent and independent manner during oxidative stress. Abbreviations: dipeptidyl peptidase-IV (DPP-IV), glucagon-like peptide-1 (GLP-1), Reactive Oxygen Species (ROS), Reactive Nitrogen Species (RNS).

Nowadays, plants/natural products have provided a new source of therapeutic treatment for T2DM and play a significant role in the primary health care of more than 80% of the world’s population. In the last few years, much work has been done in the field of plant derived bioactive compounds [79,80,81]. Figure 3 represents the role of DPP-IV inhibitors along with antioxidant properties, which protect β-cells from oxidative stress-induced damage. Natural occurring DPP-IV inhibitors from plants and animals sources that may be alkaloids, phenolic acid, steroids, flavonoids, peptides, amino acid polysaccharides, peptidoglycan, and glycopeptides etc.; all these compounds contain antioxidant properties reported in various research articles. As a result, it is possible to conclude that these molecules have dual nature DPP-IV inhibition activities as well as antioxidant properties.

Furthermore, DPP-IV inhibitors help enhance insulin secretion from β-cells by inhibiting the DPP-IV enzyme and increasing GLP-1 circulation in the body. Recent studies have reported the presence of protease inhibitors, such as alkaloids, flavonoids, glycosides, phenolic acids, polysaccharides, peptidoglycan, glycopeptides, and steroids, that act as DPP-IV inhibitors, along with their antioxidant properties [17,82,83,84,85]. Table 2 presents efficacious DPP-IV inhibitors with its inhibition activity (percentage/IC_50_) from natural sources, their active parts, and medicinal uses.

## 8. Conclusions

Incretin/gut hormones have increased rapidly worldwide over the past few decades. Both GLP-1 and GIP are secreted from gut cells to enhance pancreatic β-cell mass and function. The roles of these peptides are very proficient in reducing glycosylated hemoglobin (HbA1c) and maintaining glucose homeostasis. DPP-IV inhibitors are also acceptable therapeutics and include vildagliptin, sitagliptin, and many others. Mechanistically, DPP-IV inhibitors block the activity of enzyme, to increase the half-life of GLP-1 to normal levels in the blood plasma, and this helps recover β-cell function, improve insulin secretion, and curb glucagon secretion by α-cells. Prior investigations have revealed that the primary clinical approach for DPP-IV inhibitors with antioxidant capacities involves front-line treatment, because of their capability, safety, and acceptability. DPP-IV inhibitors also improve the metabolic system (as measured by the lowering of blood glucose) without causing hypoglycemia.

The antidiabetic effects of bioactive compounds from plants and animal sources can be associated with a mixture of phytochemicals or single compounds. This review focuses on the findings of researchers and health professionals who are engaged in the field of anti-diabetic drugs. Certain synthetic inhibitors, such as gliptin family sitagliptin, vildagliptin, and natural inhibitors, include bioactive isolated compounds, and synthetic inhibitors can also include fractions such as alkaloids, phenolic acids, flavonoids, steroids, saponins, and glycosides of DPP-IV. These compounds play a major role in suppressing oxidative stress by their antioxidant potential. During diabetes condition oxidative stress generated as to overcome this situation DPP-IV inhibiters along with antioxidants play the important role to increase the insulin secretion by increasing the half-life of GLP-1 as well as antioxidant molecules help to scavenging free radicals so that oxidative effect on β cell will be minimized. Nowadays, naturally occurring inhibitors have been increasingly focused on medicinal purposes because of their non-toxic nature, fewer side effects, and easy access to the public. Furthermore, the discovery of new natural DPP-IV based antidiabetic drugs has shown great promise. There are experimental differences between DPP-IV inhibitors concerning dosing frequency, dose quantity, and their capability. Long-term acquired clinical trials will reveal whether these compound-related structural characteristics lead to clinically relevant differences. DPP-IV inhibitors, along with their antioxidant nature, may influence the immune system and its function; therefore, a longer duration is required for their safety and effectiveness evaluations. DPP-IV inhibitors will provide a better solution for the treatment of T2DM in our society.

## Figures and Tables

**Table 1:** Currently used DPP-IV inhibitors approved by the respective country or which are undergoing clinical trials.

Generic Name	Country	Brand Name
Sitagliptin	Europe, US, Japan	Januvia
Vildagliptin	Europe, US, Japan	Galvus, Equa
Saxagliptin	Europe, Japan	Onglyza
Linagliptin	Europe, US	Trajentra, Tradjenta, Trazenta
Alogliptin	Europe, US, Japan	Vipidia, Nesina
Anagliptin	Japan	Suiny
Teneligliptin	Japan	Tenelia
Gemigliptin	Korea	Zemiglo
Omarigliptin	Japan	Marizev
Gosogliptin	Russia	Pfizer
Denagliptin	USA, Finland	Glaxo
Melogliptin	Europe, US, Japan	Glenmark
Trelagliptin	Europe, US	Takeda
Retagliptin	China	---------
Evogliptin	Korea	Suganon
Carmegliptin	Switzerland	--------

**Table 2:** DPP-IV inhibitors from various natural sources, bioactive molecules or parts and their inhibition activities.

Plant Name/Natural Sources	Part of Plant/Bioactive Components Used	Medicinal/as Food Use	* DPP-IV Inhibition Activity %/IC_50_	References
*Azadirachta indica*	Leaves	Arthritis, anxiety, trouble sleeping, anti-diabetes	17.78%	[86]
*Physalis angulata* L.	Leaves	Peroxidation, cardiotonic, hypotensive, anti-diabetes	13.94%	[87]
*Aspergillus oryzae*	tetrahydroxyisoquinoline derivative(WYK-1)	Used as food	IC50-6.98 μM	[88]
*Berberis aristata*	Bark	Arthritis, anxiety, trouble sleeping, anti-diabetes	65%	[89]
*Helichrysum rubicundum*	Flower	Anti-lipid, peroxidation, cardiotonic, hypotensive, antidegenerative, anti-diabetes	50.2%	[79]
*Hippophaёrhamnoides*	Leaves	Excessive menstruation and diarrhea	80.5%	
*Origanum vulgare*	Leaves/ Flower	Regulate blood sugar & lipid, diarrhea, cold	44.9%	
*Rubuscaesius*	Leaves	Antidiabetes, Blood Disorder, Respiratory Disorder, Piles	72.7%	
*Zea mays*	Kernels	Anti-diabetes, Arthritis, anxiety, sleeping trouble, anti-diabetes	22.2%	
*Melilotus officinalis*	Leaves	Hypertension, antioxidants prevent aging, arteriosclerosis	40.5%	
*Chamomillaerecutita*	Leaves	Diarrhea and excessive menstruation	36.6%	
*Hypericumperforatum*	Seed	Hypertension, antioxidants prevent aging	47.7%	
*Castanospermum australe*	Seed	Anti-diabetes, Arthritis, anxiety, trouble sleeping, antidiabetic	68.0	[20]
*Citrus sinensis*	Fruit Peel	Hyperglycimia, anti-inflammation& antioxidant	Not mention	[90]
*Amaranthus hypochondriacus*	Seed protein	Excessive menstruation and diarrhea	50%	[60]
*Phalariscanariensis*	Seed	Hypertension, antioxidants preventing aging and arteriosclerosis	43.5%	[91]
*Trigonella foenum*	Seed	Reproductive or sin problems, antidiabetic and ulcers.	72.6 ± 0.8	[92]
*Withania somnifera*	Root powder	Anxiety, sleeping problem, antioxidant, anti-diabetic	88.35 ± 0.8	
*Ocimum sanctum*	Leaves	Anti-cancer, heptoprotective Anti-diabetic, anti-microbial, and cardio-protective	66.81 ± 0.05	[83]
*Momardica charantia*	Fruits	Respiratory and blood disorder, Anti-diabetic, piles, antioxidant.	53.25 ± 0.04	
LPVPQ Peptide & IPM	Milk	Antioxidant, Anti-microbial, ACE inhibitor	IC50 43.8 μΜ & 69.3 μM	[30]
*Aloe barbadensis*	Leaves	Anti-microbial, anti-diabetic, anti-ulcer, hepatoprotactive, Anti-cancer.	IC50 2.71 mg/mL	[55]
*Terminalia arjuna*	Bark	Cardiotonic, anti-diabetic, Anti-dysenteric, anti-pyretic	83.39%	[93]
*Commiphora mukul*	Gum-resin	Anti-inflammation, antidiabetic	92.97%	
*Gymnema sylvestre*	Leaves	Anti-diabetic, lower blood pressure and cholesterol	16.98%	
*Morinda citrifolia*	Fruits	Reduce blood pressure, anti-diabetic, anti-depression, anxiety	24.64%	
*Emblica officinalis*	Fruits	Antioxidant, anti-inflammation,	85.95%	
*Arachishypogaea*	Seed	Anti-lipid, peroxidation, cardiotonic, hypotensive, antidegenerative, anti-diabetes	51%	[94]
*Senna nigricans*	Leaves	Skin irritations, anti-diabetes, ulcers,	57%	
*Solanum incanum*	Fruit	Arthritis, anxiety, trouble sleeping, anti-diabetes	68.1%	
*Ziziphusmauritiana*	Root	Anti-cancer, anti-diabetic, anti-microbial, hepatoprotective, cardioprotective, antiemetic	56.6%	
FSD & WSG	Barbel muscle protein	Used as food and ornamental	IC 50 1.96 mg/mL	[95]
*Abelmoschus esculentus*	Fruits	Anti-spasmodic, diuretic, anti-diabetic,	------	[96]
*Mangifera indica*	Leaves	Antioxidant, cardiotonic, hypotension, anti-diabetic	68.24%	[80,97]
*Origanum vulgare*	Leaves	Hypotension, anti-degenerative, anti-diabetic.	44.9%	[80]
*Menthapiperita*	Leaves	Anti-diabetic, anti-cancer, hepatoprotective, anti-microbial, antiemetic	38.2%	
*Lagerstroemia loudonii*	Leaves	Hypertension, antioxidants	60.22%	
LPVP & MPVQA	Camel milk	Used as food	IC50- 87.0 μM & 93.3 μM	[98]
*Camellia sinensis*	Leaves	Hypertension, antioxidants prevent aging, arteriosclerosis	50.47%	[99]
*Caesapinia sappan*	Heartwood	Antioxidant, anti-inflammatory, hepatoprotective, cytotoxic & hypoglycemia inhibition activity	84.25%	[100]
*Cinchona officinalis*	Stem bark	Used as blood vessel disorder, Increase juice, increase production of digest	62.95%	
*Elephantopusscaber*	Roots	Treatment for pain, edema, fever and cold cough.	48.17%	
*Muntingiacalabura*	Leaves	Antispasmodic, headache, cold cough	74.12%	
*Foeniculum vulgare*	Seeds	Treatment for respiratory, blood pressure, digestive problem diuretic.	46.15%	
*Morus nigra*	Stem bark	Anti-bacterial, anti-diabetic, hypertension	51%	
*Phyllanthns niuri*	Aerial parts	Treatment for liver disease, anti-cancer, anti-diabetic, anti-hypertensive	70.48%	
*Psidium guajava*	Leaves	Treatment for diarrhea, dysentery, pain relief, Anti-diabetic, hypertension.	66.11%	
*Rheum palmatum*	Roots	Fever and edema, anti-inflammatory	72.67%	
*Vernania amygdalina*	Leaves	Anti-malaria, anti-diabetic kidney disease, anti-hypertensive	50.2%	
*Prosopis cineraria*	Pod	used for treatment of asthma, bronchitis skin problem	64.8%	[101]
*Garlic bulb*	Bulb	Antioxidant, anti-hypertensive, anti-inflammatory, anti-diabetic.	IC50- 70.88 μg/ml	[102]
*subtilisin-flavourzyme*	enebriomolitor hydrolysates	Antioxidant, anti-hypertensive.	IC50- 2.89mg/mL	[103]
*Castanea mollissima Blume*	Chestnut Inner skin	Nuts used as food and antioxidant.	IC50 1.14 μg/ml	[104]
*Caprosaper*	Protein hydrolysate IPVDM & IPV	Ornamental used only	IC50- 21.72 μM & 5.61 μM	[105]
*Arthrospira platensis* (spirulina)	C-phycocyanin (C-PC) and allophycocyanin (APC)	Anti-apoptotic, hypolipidemic, anti-inflammatory	95.8%	[106]
α-lactalbumin-rich whey protein	LDQWLCEKL	Used as source of food	131 μM	[107]
*Hibiscus rosa-sinensis*	Leaves	Detoxifier, anti-fertility, anti-cancer, anti-hypertension, and cardio-protective effect	60%	[108]
*Aloe vera*	dipyrrole derivative	Used as anti-diabetic, skin problem,	IC50 – 8.59 nM	[109]
*Quercetin & Coumarin*	Flavonoids (bioactive compounds)	Anti-hyperglycemic and antioxidant	IC50-4.02 &54.8 nMol/ml	[84]
*Moringa oleifera* *Lam.*	Leaves	Anti-inflammatory, anti-diabetic, hepatoprotective	IC50- 798 nM	[110]

* The higher percentage represents higher the inhibition activity of molecules. The percentage of inhibition was calculated. Data are expressed as Mean ± Standard deviation, *n* = 3 replicate. % inhibition = absorbance of control − absorbance of inhibitor/absorption of control × 100.

## References

[1] White J.R. (2014). A brief history of the development of diabetes medications. Diabetes Spectr..

[2] Chiasson J.-L., Josse R.G., Gomis R., Hanefeld M., Karasik A., Laakso M., The STOP-NIDDM Trial Research Group (2003). Acarbose treatment and the risk of cardiovascular disease and hypertension in patients with impaired glucose tolerance. JAMA.

[3] Yadav D., Tiwari A., Mishra M., Subramanian S.S., Baghel U.S., Mahajan S., Bisen P.S., Prasad G.B. (2014). Anti-hyperglycemic and anti-hyperlipidemic potential of a polyherbal preparation “diabegon” in metabolic syndrome subject with type 2 diabetes. Afr. J. Tradit. Complement Altern. Med..

[4] Krentz A.J., Bailey C.J. (2005). Oral antidiabetic agents: Current role in type 2 diabetes mellitus. Drugs.

[5] Pinchevsky Y., Butkow N., Raal F.J., Chirwa T., Rothberg A. (2020). Demographic and clinical factors associated with development of type 2 diabetes: A review of the literature. Int. J. Gen. Med..

[6] International Diabetes Federation (2019). IDF Diabetes Atlas.

[7] Bodmer M., Meier C., Krähenbühl S., Jick S.S., Meier C.R. (2008). Metformin, sulfonylureas, or other anti-diabetes drugs and the risk of lactic acidosis or hypoglycemia: A nested case-control analysis. Diabetes Care.

[8] Forst T., Bramlage P. (2014). Vildagliptin, a dpp-4 inhibitor for the twice-daily treatment of type 2 diabetes mellitus with or without metformin. Expert Opin. Pharmacother..

[9] Heine R.J., Van Gaal L.F., Johns D., Mihm M.J., Widel M.H., Brodows R.G., The GWAA Study Group (2005). Exenatide versus insulin glargine in patients with suboptimally controlled type 2 diabetes: A randomized trial. Ann. Intern. Med..

[10] Maritim A.C., Sanders R.A., Watkins J.B. (2003). Diabetes, oxidative stress, and antioxidants: A review. J. Biochem. Mol. Toxicol..

[11] Abedini A., Schmidt A.M. (2013). Mechanisms of islet amyloidosis toxicity in type 2 diabetes. FEBS Lett..

[12] Ram H., Singh A.-K. (2020). Improvements in insulin resistance and β-cells dysfunction by ddp-4 inhibition potential of *Withania Somnifera* (L.) dunal root extract in type 2 diabetic rat. Biointerface Res. Appl. Chem..

[13] Müller T., Finan B., Bloom S., D’Alessio D., Drucker D., Flatt P., Fritsche A., Gribble F., Grill H., Habener J. (2019). Glucagon-like peptide 1 (glp-1). Mol. Metab..

[14] Gribble F.M., Reimann F. (2021). Metabolic messengers: Glucagon-like peptide 1. Nat. Metab..

[15] Silveira S.T., Martínez-Maqueda D., Recio I., Hernández-Ledesma B. (2013). Dipeptidyl peptidase-iv inhibitory peptides generated by tryptic hydrolysis of a whey protein concentrate rich in β-lactoglobulin. Food Chem..

[16] Ng V.W.S., Glasg F. (2007). Dipeptidyl peptidase (dpp) -iv inhibitor: A novel class of oral anti-hyperglycemic agents. Clin. Pharmacol. Ther..

[17] Paliwal G., Sharma A., Upadhyay N., Das M., Tiwari A. (2015). Therapeutic stimulation of glp-1 protein by implementing in silico to in vitro approach for type-2 diabetes treatment. Middle East J. Sci. Res..

[18] Lammi C., Zanoni C., Arnoldi A., Vistoli G. (2016). Peptides derived from soy and lupin protein as dipeptidyl-peptidase iv inhibitors: In vitro biochemical screening and in silico molecular modeling study. J. Agric. Food Chem..

[19] Mowla A., Alauddin M., Rahman M.A., Ahmed K. (2009). Antihyperglycemic effect of *trigonella foenum-graecum* (fenugreek) seed extract in alloxan-induced diabetic rats and its use in diabetes mellitus: A brief qualitative phytochemical and acute toxicity test on the extract. Afr. J. Tradit. Complement Altern. Med..

[20] Bharti S.K., Krishnan S., Kumar A., Rajak K.K., Murari K., Bharti B.K., Gupta A.K. (2012). Antihyperglycemic activity with dpp-iv inhibition of alkaloids from seed extract of *castanospermum australe*: Investigation by experimental validation and molecular docking. Phytomedicine.

[21] Jadav P., Bahekar R., Shah S.R., Patel D., Joharapurkar A., Kshirsagar S., Jain M., Shaikh M., Sairam K.V.V.M. (2012). Long-acting peptidomimetics based dpp-iv inhibitors. Bioorg. Med. Chem. Lett..

[22] Hopsu-Havu V.K., Glenner G.G. (1966). A new dipeptide naphthylamidase hydrolyzing glycyl-prolyl-beta-naphthylamide. Histochemie.

[23] Barnett A. (2006). Dpp-4 inhibitors and their potential role in the management of type 2 diabetes. Int. J. Clin. Pract..

[24] Varona A., Blanco L., Perez I., Gil J., Irazusta J., López J.I., Candenas M.L., Pinto F.M., Larrinaga G. (2010). Expression and activity profiles of DPP IV/CD26 and NEP/CD10 glycoproteins in the human renal cancer are tumor-type dependent. BMC Cancer.

[25] Leiting B., Pryor K.D., Wu J.K., Marsilio F., Patel R.A., Craik C.S., Ellman J.A., Cummings R.T., Thornberry N.A. (2003). Catalytic properties and inhibition of proline-specific dipeptidyl peptidases ii, iv and vii. Biochem. J..

[26] Lim S.W., Jin L., Piao S.G., Chung B.H., Yang C.W. (2015). Inhibition of dipeptidyl peptidase iv protects tacrolimus-induced kidney injury. Lab. Invest..

[27] Püschel G., Mentlein R., Heymann E. (1982). Isolation and characterization of dipeptidyl peptidase iv from human placenta. Eur. J. Biochem..

[28] Davy A., Thomsen K.K., Juliano M.A., Alves L.C., Svendsen I., Simpson D.J. (2000). Purification and characterization of barley dipeptidyl peptidase iv. Plant Physiol..

[29] Nongonierma A.B., FitzGerald R.J. (2013). Inhibition of dipeptidyl peptidase iv (dpp-iv) by proline containing casein-derived peptides. J. Funct. Foods.

[30] Nongonierma A.B., FitzGerald R.J. (2014). Susceptibility of milk protein-derived peptides to dipeptidyl peptidase iv (dpp-iv) hydrolysis. Food Chem..

[31] Lee D.-S., Lee E.-S., Alam M., Jang J.-H., Lee H.-S., Oh H., Kim Y.-C., Manzoor Z., Koh Y.-S., Kang D.-G. (2016). Soluble dpp-4 up-regulates toll-like receptors and augments inflammatory reactions, which are ameliorated by vildagliptin or mannose-6-phosphate. Metabolism.

[32] Ishibashi Y., Matsui T., Maeda S., Higashimoto Y., Yamagishi S.-I. (2013). Advanced glycation end products evoke endothelial cell damage by stimulating soluble dipeptidyl peptidase-4 production and its interaction with mannose 6-phosphate/insulin-like growth factor ii receptor. Cardiovasc. Diabetol..

[33] Ikeda T., Kumagai E., Iwata S., Yamakawa A. (2013). Soluble cd26/dipeptidyl peptidase iv enhances the transcription of il-6 and tnf-α in thp-1 cells and monocytes. PLoS ONE.

[34] Deacon C.F., Ahrén B., Holst J.J. (2004). Inhibitors of dipeptidyl peptidase iv: A novel approach for the prevention and treatment of type 2 diabetes?. Expert Opin. Investig. Drugs.

[35] Cantini G., Mannucci E., Luconi M. (2016). Perspectives in glp-1 research: New targets, new receptors. Trends Endocrinol. Metab..

[36] Terrill S.J., Jackson C.M., Greene H.E., Lilly N., Maske C.B., Vallejo S., Williams D.L. (2016). Role of lateral septum glucagon-like peptide 1 receptors in food intake. Am. J. Physiol. Integr. Comp. Physiol..

[37] Franz M.J., Bantle J.P., Beebe C.A., Brunzell J.D., Chiasson J.-L., Garg A., Holzmeister L.A., Hoogwerf B., Mayer-Davis E., Mooradian A.D. (2002). Evidence-based nutrition principles and recommendations for the treatment and prevention of diabetes and related complications. Diabetes Care.

[38] Gupta V. (2013). Glucagon-like peptide-1 analogues: An overview. Indian J. Endocrinol. Metab..

[39] Pham-Huy L.A., He H., Pham-Huy C. (2008). Free radicals, antioxidants in disease and health. Int. J. Biomed. Sci. IJBS.

[40] Pizzino G., Irrera N., Cucinotta M., Pallio G., Mannino F., Arcoraci V., Squadrito F., Altavilla D., Bitto A. (2017). Oxidative stress: Harms and benefits for human health. Oxid. Med. Cell. Longev..

[41] Phaniendra A., Jestadi D.B., Periyasamy L. (2015). Free radicals: Properties, sources, targets, and their implication in various diseases. Indian J. Clin. Biochem..

[42] Kumar H., Lim H.-W., More S.V., Kim B.-W., Koppula S., Kim I.S., Choi D.-K. (2012). The role of free radicals in the aging brain and parkinson’s disease: Convergence and parallelism. Int. J. Mol. Sci..

[43] Yadav D., Mishra M., Joseph A.Z., Subramani S.K., Mahajan S., Singh N., Bisen P.S., Prasad G.B. (2015). Status of antioxidant and lipid peroxidation in type 2 diabetic human subjects diagnosed with and without metabolic syndrome by using ncep-atpiii, idf and who criteria. Obes. Res. Clin. Pract..

[44] Pacher P., Beckman J.S., Liaudet L. (2007). Nitric oxide and peroxynitrite in health and disease. Physiol. Rev..

[45] Di Meo S., Reed T.T., Venditti P., Victor V.M. (2016). Role of ros and rns sources in physiological and pathological conditions. Oxid. Med. Cell. Longev..

[46] Bardaweel S.K., Gul M., Alzweiri M., Ishaqat A., Alsalamat H.A., Bashatwah R.M. (2018). Reactive oxygen species: The dual role in physiological and pathological conditions of the human body. Eurasian J. Med..

[47] Guo C., Sun L., Chen X., Zhang D. (2013). Oxidative stress, mitochondrial damage and neurodegenerative diseases. Neural Regen. Res..

[48] Rahman K. (2007). Studies on free radicals, antioxidants, and co-factors. Clin. Interv. Aging.

[49] Kurutas E.B. (2016). The importance of antioxidants which play the role in cellular response against oxidative/nitrosative stress: Current state. Nutr. J..

[50] Bashir H.S., Mohammed A.M., Magsoud A.S., Shaoub A.M. (2013). Isolation of three flavonoids from *withania somnifera* leaves (solanaceae) and their antimicrobial activities. J. For. Prod. Ind..

[51] Mustafa R., Hamid A.A., Mohamed S., Bakar F.A. (2010). Total phenolic compounds, flavonoids, and radical scavenging activity of 21 selected tropical plants. J. Food Sci..

[52] Kaurinovic B., Vastag D. (2019). Flavonoids and phenolic acids as potential natural antioxidants. Antioxidants.

[53] Katare C., Saxena S., Agrawal S., Joseph A.Z., Subramani S.K., Yadav D., Singh N., Bisen P.S., Prasad G.B. (2014). Lipid-lowering and antioxidant functions of bottle gourd (lagenaria siceraria) extract in human dyslipidemia. J. Evid. Based Complementary Altern. Med..

[54] Mir M.A., Sawhney S.S., Jassal M.M.S. (2013). Qualitative and quantitative analysis of phytochemicals of taraxacum officinale. Wudpecker J. Pharm. Pharmocology.

[55] Raja C.P., Venkataraman K. (2016). Aloe vera phytochemicals inhibits dipeptidyl peptidase iv (dpp-iv), an anti-diabetic target. Int. J. Pharm. Bio. Sci..

[56] Mojzer E.B., Hrnčič M.K., Škerget M., Knez Ž., Bren U. (2016). Polyphenols: Extraction methods, antioxidative action, bioavailability and anticarcinogenic effects. Molecules.

[57] Li A.-N., Li S., Zhang Y.-J., Xu X.-R., Chen Y.-M., Li H.-B. (2014). Resources and biological activities of natural polyphenols. Nutrients.

[58] Pandey K.B., Rizvi S.I. (2009). Plant polyphenols as dietary antioxidants in human health and disease. Oxid. Med. Cell. Longev..

[59] Kumar N., Goel N. (2019). Phenolic acids: Natural versatile molecules with promising therapeutic applications. Biotechnol. Rep..

[60] Velarde-Salcedo A.J., Barrera-Pacheco A., Lara-Gonzalez S., Montero-Morán G.M., Díaz-Gois A., DE Mejia E., de la Rosa A.P.B. (2013). In vitro inhibition of dipeptidyl peptidase iv by peptides derived from the hydrolysis of amaranth (*amaranthus hypochondriacus* L.) proteins. Food Chem..

[61] Green A.D., Vasu S., Moffett R.C., Flatt P.R. (2016). Biochimie co-culture of clonal beta cells with glp-1 and glucagon-secreting cell line impacts on beta cell insulin secretion, proliferation and susceptibility to cytotoxins. Biochimie.

[62] Janssen P., Rotondo A., Mulé F., Tack J. (2013). Review article: A comparison of glucagon-like peptides 1 and 2. Aliment. Pharmacol. Ther..

[63] Seino Y., Fukushima M., Yabe D. (2010). Gip and glp-1, the two incretin hormones: Similarities and differences. J. Diabetes Investig..

[64] Brubaker P.L., Drucker D.J. (2004). Minireview: Glucagon-like peptides regulate cell proliferation and apoptosis in the pancreas, gut, and central nervous system. Endocrinology.

[65] Cabou C., Burcelin R. (2011). GLP-1, the gut-brain, and brain-periphery axes. Rev Diabet Stud..

[66] Qiao Q., Grandy S., Hiller J., Kostev K. (2016). Clinical and patient-related variables associated with initiating glp-1 receptor agonist therapy in type 2 diabetes patients in primary care in Germany. PLoS ONE.

[67] Kulve J.S.T., Veltman D.J., Van Bloemendaal L., Barkhof F., Deacon C.F., Holst J.J., Konrad R.J., Sloan J.H., Drent M.L., Diamant M. (2015). Endogenous glp-1 mediates postprandial reductions in activation in central reward and satiety areas in patients with type 2 diabetes. Diabetologia.

[68] Fujita Y., Wideman R.D., Asadi A., Yang G., Baker R., Webber T., Zhang T., Wang R., Ao Z., Warnock G.L. (2010). Glucose-dependent insulinotropic polypeptide is expressed in pancreatic islet α-cells and promotes insulin secretion. Gastroenterology.

[69] Young A.A., Gedulin B.R., Bhavsar S., Bodkin N., Jodka C., Hansen B., Denaro M. (1999). Glucose-lowering and insulin-sensitizing actions of exendin-4: Studies in obese diabetic (ob/ob, db/db) mice, diabetic fatty zucker rats, and diabetic rhesus monkeys (*macaca mulatta*). Diabetes.

[70] Gallwitz B. (2011). Glp-1 agonists and dipeptidyl-peptidase iv inhibitors. Handb. Exp. Pharmacol..

[71] Łabuzek K., Kozłowski M., Szkudłapski D., Sikorska P., Kozłowska M., Okopień B. (2013). Incretin-based therapies in the treatment of type 2 diabetes—More than meets the eye?. Eur. J. Intern. Med..

[72] Mari A., Sallas W.M., He Y.L., Watson C., Ligueros-Saylan M., Dunning B.E., Deacon C.F., Holst J.J., Foley J.E. (2005). Vildagliptin, a dipeptidyl peptidase-iv inhibitor, improves model-assessed beta-cell function in patients with type 2 diabetes. J. Clin. Endocrinol. Metab..

[73] Trujillo J.M., Nuffer W., Ellis S.L. (2015). Glp-1 receptor agonists: A review of head-to-head clinical studies. Ther. Adv. Endocrinol. Metab..

[74] Singh T.P., Vangaveti V.N., Malabu U.H. (2015). Dipeptidyl peptidase-4 inhibitors and their potential role in the management of atherosclerosis--a review. Diabetes Metab. Syndr..

[75] Yogisha S., Raveesha K.A. (2010). Dipeptidyl Peptidase IV inhibitory activity of *Mangifera indica*. J. Nat. Prod..

[76] Ta N.N., Schuyler C.A., Li Y., Lopes-Virella M.F., Huang Y. (2011). Dpp-4 (cd26) inhibitor alogliptin inhibits atherosclerosis in diabetic apolipoprotein e-deficient mice. J. Cardiovasc. Pharmacol..

[77] Hiromura M., Nohtomi K., Mori Y., Kataoka H., Sugano M., Ohnuma K., Kuwata H., Hirano T. (2018). Caveolin-1, a binding protein of cd26, is essential for the anti-inflammatory effects of dipeptidyl peptidase-4 inhibitors on human and mouse macrophages. Biochem. Biophys. Res. Commun..

[78] Terasaki M., Yashima H., Mori Y., Saito T., Matsui T., Hiromura M., Kushima H., Osaka N., Ohara M., Fukui T. (2020). A dipeptidyl peptidase-4 inhibitor inhibits foam cell formation of macrophages in type 1 diabetes via suppression of cd36 and acat-1 expression. Int. J. Mol. Sci..

[79] Mardanyan S., Sharoyan S., Antonyan A., Zakaryan N. (2011). Dipeptidyl peptidase iv and adenosine deaminase inhibition by Armenian plants and antidiabetic drugs. Int. J. Diabetes Metab..

[80] Saidu Y., Muhammad S., Yahaya A., Onu A., Mohammed I., Muhammad L. (2017). In vitro screening for protein tyrosine phosphatase 1b and dipeptidyl peptidase iv inhibitors from Nigerian medicinal plants. J. Intercult. Ethnopharmacol..

[81] Singh A.K., Jatwa R., Purohit A., Ram H. (2017). Synthetic and phytocompounds based dipeptidyl peptidase-iv (dpp-iv) inhibitors for therapeutics of diabetes. J. Asian Nat. Prod. Res..

[82] Mallikharjuna P.B., Rajanna L.N., Seetharam Y.N., Sharanabasappa G.K. (2007). Phytochemical studies of strychnos potatorum l.F.—A medicinal plant. E J. Chem..

[83] Singh A.-k., Jatwa R., Joshi J. (2014). Cytoprotective and dipeptidyl peptidase-iv (dpp-iv/cd26) inhibitory roles of *ocimum sanctum* and *momordica charantia* extract. Asian J. Pharm. Clin. Res..

[84] Singh A.K., Patel P.K., Choudhary K., Joshi J., Yadav D., Jin J.O. (2020). Quercetin and coumarin inhibit dipeptidyl peptidase-iv and exhibits antioxidant properties: In silico, in vitro, ex vivo. Biomolecules.

[85] Lee E.S., Kim H.M., Kang J.S., Lee E.Y., Yadav D., Kwon M.H., Kim Y.M., Kim H.S., Chung C.H. (2016). Oleanolic acid and n-acetylcysteine ameliorate diabetic nephropathy through reduction of oxidative stress and endoplasmic reticulum stress in a type 2 diabetic rat model. Nephrol. Dial. Transplant..

[86] Krishnaiah D., Devi T., Bono A., Sarbatly R. (2009). Studies on phytochemical constituents of six malaysian medicinal plants. J. Med. Plants Res..

[87] Ameh G.I., Eze C.S. (2010). Phytochemical constituents of some Nigerian plants. BioResearch.

[88] Imamura K., Tsuyama Y., Hirata T. (2010). Identification and characterization of a novel fermented substance produced by edible *aspergillus oryzae* ao-1 that inhibits dpp-iv activity. J. Biosci. Bioeng..

[89] Chakrabarti R., Bhavtaran S., Narendra P., Varghese N. (2011). Journal of natural products dipeptidyl peptidase- iv inhibitory activity of *Berberis aristata*. Nat. Prod..

[90] Parmar H.S., Jain P., Chauhan D.S., Bhinchar M.K., Munjal V., Yusuf M., Choube K., Tawani A., Tiwari V., Manivannan E. (2012). Dpp-iv inhibitory potential of naringin: An in silico, in vitro and in vivo study. Diabetes Res. Clin. Pract..

[91] Estrada-Salas P.A., Montero-Morán G.M., Martínez-Cuevas P.P., González C., Barba de la Rosa A.P. (2014). Characterization of antidiabetic and antihypertensive properties of canary seed (*Phalaris canariensis* L.) peptides. J. Agric. Food Chem..

[92] Singh A.K., Joshi J., Jatwa R. (2013). Dipeptidyl peptidase iv (dpp-iv/cd26) inhibitory and free radical scavenging potential of *w. Somnifera* and *t. Foenum-graecum* extract. Int. J. Phytomed..

[93] Borde M.K., Mohanty I.R., Suman R.K., Deshmukh Y.A. (2016). Dipeptidyl peptidase-iv inhibitory activities of medicinal plants: *Terminalia arjuna*, *Commiphora mukul*, *Gymnema sylvestre*, *Morinda citrifolia*, *Emblica officinalis*. Asian J. Pharma. Clin. Res..

[94] Riyanti S., Suganda A.G., Sukandar E.Y. (2016). Dipeptidyl peptidase-iv inhibitory activity of some indonesian medicinal plants. Asian J. Pharma. Clin. Res..

[95] Sila A., Alvarez O.M., Haddar A., Frikha F., Dhulster P., Nedjar-Arroume N., Bougatef A. (2016). Purification, identification and structural modelling of dpp-iv inhibiting peptides from barbel protein hydrolysate. J. Chromatogr. B Anal. Technol. Biomed. Life Sci..

[96] Huang C.N., Wang C.J., Lee Y.J., Peng C.H. (2017). Active subfractions of *abelmoschus esculentus* substantially prevent free fatty acid-induced ß cell apoptosis via inhibiting dipeptidyl peptidase-4. PLoS ONE.

[97] Kinghorn A.D., Pan L., Fletcher J.N., Chai H. (2011). The Relevance of Higher Plants in Lead Compound Discovery Programs. J. Nat. Prod..

[98] Nongonierma A.B., Paolella S., Mudgil P., Maqsood S., FitzGerald R.J. (2018). Identification of novel dipeptidyl peptidase iv (dpp-iv) inhibitory peptides in camel milk protein hydrolysates. Food Chem..

[99] Ekayanti M., Sauriasari R., Elya B. (2018). Dipeptidyl peptidase iv inhibitory activity of fraction from white tea ethanolic extract (*Camellia sinensis* (L.) kuntze) ex vivo. Pharmacogn. J..

[100] Setyaningsih E.P., Saputri F.C., Mun’im A. (2019). The antidiabetic effectivity of indonesian plants extracts via dpp-iv inhibitory mechanism. J. Young Pharm..

[101] Ram H., Jaipal N., Kumar P., Deka P., Kumar S., Kashyap P., Kumar S., Singh B.P., Alqarawi A.A., Hashem A. (2019). Dual inhibition of dpp-4 and cholinesterase enzymes by the phytoconstituents of the ethanolic extract of prosopis *cineraria* pods: Therapeutic implications for the treatment of diabetes-associated neurological impairments. Curr. Alzheimer Res..

[102] Kalhotra P., Chittepu V.C.S.R., Osorio-Revilla G., Gallardo-Velazquez T. (2020). Phytochemicals in garlic extract inhibit therapeutic enzyme dpp-4 and induce skeletal muscle cell proliferation: A possible mechanism of action to benefit the treatment of diabetes mellitus. Biomolecules.

[103] Pino F.R., Gálvez A.R.P., Carpio F.J.E., Guadix E.M. (2020). Evaluation of tenebrio molitor protein as source of peptides modulating physiological processes. Food Funct..

[104] Zhang Y., Yang Z., Liu G., Wu Y., Ouyang J. (2020). Inhibitory effect of chestnut (*Castanea mollissima* Blume) inner skin extract on the activity of α-amylase, α-glucosidase, dipeptidyl peptidase iv and in vitro digestibility of starches. Food Chem..

[105] Harnedy-Rothwell P.A., McLaughlin C.M., O’Keeffe M.B., Le Gouic A.V., Allsopp P.J., McSorley E.M., Sharkey S., Whooley J., McGovern B., O’Harte F.P. (2020). Identification and characterisation of peptides from a boarfish (*Capros aper*) protein hydrolysate displaying in vitro dipeptidyl peptidase-iv (dpp-iv) inhibitory and insulinotropic activity. Food Res. Int..

[106] Li Y., Aiello G., Bollati C., Bartolomei M., Arnoldi A., Lammi C. (2020). Phycobiliproteins from *arthrospira platensis* (spirulina): A new source of peptides with dipeptidyl peptidase-iv inhibitory activity. Nutrients.

[107] Jia C.-L., Hussain N., Ujiroghene O.J., Pang X.-Y., Zhang S.-W., Lu J., Liu L., Lv J.-P., Obaroakpo J.U. (2020). Generation and characterization of dipeptidyl peptidase-iv inhibitory peptides from trypsin-hydrolyzed α-lactalbumin-rich whey proteins. Food Chem..

[108] Ansari P., Azam S., Hannan J.M.A., Flatt P.R., Abdel Wahab Y.H.A. (2020). Anti-hyperglycaemic activity of *h. Rosa-sinensis* leaves is partly mediated by inhibition of carbohydrate digestion and absorption, and enhancement of insulin secretion. J. Ethnopharmacol..

[109] Prasannaraja C., Kamalanathan A.S., Vijayalakshmi M.A., Venkataraman K. (2020). A dipyrrole derivative from aloe vera inhibits an anti-diabetic drug target dipeptidyl peptidase (dpp)-iv in vitro. Prep. Biochem. Biotechnol..

[110] Yang Y., Shi C.Y., Xie J., Dai J.H., He S.L., Tian Y. (2020). Identification of potential dipeptidyl peptidase (dpp)-iv inhibitors among *Moringa oleifera* phytochemicals by virtual screening, molecular docking analysis, adme/t-based prediction, and in vitro analyses. Molecules.

